# Health-Related Lifestyle Profiles in Healthy Adults: Associations with Sociodemographic Indicators, Dispositional Optimism, and Sense of Coherence

**DOI:** 10.3390/nu13113778

**Published:** 2021-10-25

**Authors:** Roberta Adorni, Francesco Zanatta, Marco D’Addario, Francesca Atella, Elena Costantino, Caterina Iaderosa, Giulia Petarle, Patrizia Steca

**Affiliations:** Department of Psychology, University of Milano-Bicocca, 20126 Milan, Italy; francesco.zanatta@unimib.it (F.Z.); marco.daddario@unimib.it (M.D.); f.atella@campus.unimib.it (F.A.); e.costantino1@campus.unimib.it (E.C.); c.iaderosa@campus.unimib.it (C.I.); g.petarle@campus.unimib.it (G.P.); patrizia.steca@unimib.it (P.S.)

**Keywords:** lifestyle, diet, alcohol consumption, physical activity, cigarette smoking, cardiovascular screening, gender, dispositional optimism, sense of coherence

## Abstract

Cardiovascular disease (CVD) is the leading cause of morbidity and mortality globally. Promoting healthy behaviors throughout life is an essential prevention tool. This study investigated the associations among lifestyle profiles (including diet, alcohol consumption, physical activity, cigarette smoking, and cardiovascular screening), sociodemographic factors (gender, age, education, and family history of CVDs), and psychological factors (sense of coherence and dispositional optimism). In total, 676 healthy adults (mean age = 35 years; range = 19–57; 46% male) participated in an online survey. Lifestyle profiles were identified through cluster analysis, and a multinomial logistic regression was then performed to explore their association with sociodemographic and psychological variables. Results show that men were more likely than women to belong to the lifestyle profile with the highest amount of physical activity (OR = 2.40; *p* < 0.001) and the greatest attention to cardiovascular screening (OR = 2.09; *p* < 0.01). Lower dispositional optimism was associated with the profile paying the greatest attention to cardiovascular screening (OR = 0.67; *p* < 0.05). Sense of coherence, in terms of lower comprehensibility (OR = 0.67; *p* < 0.05) and higher manageability (OR = 1.43; *p* < 0.05), was associated with the lifestyle profile characterized by an unhealthy diet, sedentary lifestyle, and nonsmoking. This study shed light on factors associated with different co-occurring health-related behaviors that should be considered in planning effective communication strategies promoting adherence to health claims.

## 1. Introduction

In recent decades, there has been substantial improvement in cardiovascular disease (CVD) management and outcomes. Despite this, CVDs remain the leading cause of morbidity and mortality globally [1,2]. It is widely recognized that the most important way to prevent CVDs is to promote a healthy lifestyle throughout life [1,2]. Therefore, in order to specifically prevent the development of the main risk factors causing CVDs later in life, the importance of encouraging a healthy lifestyle has recently emerged. This is the case not only for those who have suffered a cardiovascular event (secondary prevention) or those with multiple cardiovascular risk factors (primary prevention) but for the healthy population as well (primordial prevention) [3,4]. The European guidelines on cardiovascular disease prevention [5] state that cardiovascular risk should be considered as an increasingly universal, urgent, and emerging issue in clinical practice both in high-risk individuals and healthy populations.

For this reason, preventive strategies should be extended over the entire life span, as it is also shown that CVDs affect not only the elderly population but also the youngest. Moderation in alcohol intake, a physically active lifestyle, nonsmoking, and a diet containing little saturated fat, total fat, and sodium are crucial to decrease cardiovascular risks [1]. For example, it has been demonstrated that nutraceuticals and functional food ingredients are beneficial to cardiovascular health, representing protective factors against the overall cardiovascular risk induced by dyslipidemia [6]. Moreover, assessing the main risk factors and encouraging cardiovascular screening for healthy adults between 20 and 39 at least every 4 to 6 years and more frequently, depending on the number of risk factors, in adults aged 40 to 75 are strongly recommended [1]. Therefore, a primordial prevention framework considering such factors remains of paramount concern when aiming to pre-empt the onset of premature diseases.

Accordingly, it is essential to look at the inter-correlational nature of healthy lifestyles and behaviors. Plenty of studies have shown that multiple health behaviors have a concurrent effect and should be targeted simultaneously by adopting a clustering approach [7,8,9]. For instance, some prior works have found that smoking and alcohol consumption may co-occur with each other [8,10] and with an inappropriate diet: consumption of more fat and less fruit and vegetables [8,11]. Moreover, a sedentary lifestyle has been clustered with fatty food intake [12] and smoking habits [9]. Hence, it must be noted that, particularly in the healthy population, unhealthy behaviors do not appear randomly distributed, but instead, they tend to co-occur and interplay. The co-occurrence of unhealthy lifestyles was also found in patients with a first acute coronary event [13,14]. For instance, it was shown that a patient’s diet, physical activity, and smoking behavior could improve six months after the cardiovascular event, but those displaying multiple unhealthy behaviors can experience more difficulties in maintaining a healthier lifestyle over time. Overall, this underlines the importance of considering the inter-correlational nature of lifestyles in healthy and clinical populations and focusing on their longitudinal stability and change.

The role of psychological factors in adopting a healthy lifestyle is vastly underestimated. To date, studies revealing the associations among psychological factors, lifestyles, and subsequent cardiovascular health have focused primarily on the harmful role of anxiety and depression symptoms on both healthy [15] and medical populations [13,14,16,17]. However, accumulating evidence suggests that positive psychological resources are associated with a lower risk of developing CVDs and may promote healthy behaviors and cardiovascular health [18]. Dispositional optimism [19] and sense of coherence [20] have received remarkable attention.

Dispositional optimism is a generalized expectancy for positive rather than negative outcomes for the future [19]. Overall, previous studies have suggested that optimism is a robust predictor of diverse physical health outcomes [21] and that related higher levels are associated with a greater likelihood of engaging in healthy behaviors [18]. A recent meta-analysis showed that higher dispositional optimism was associated with a higher amount of physical activity, nonsmoking, and a healthy diet in different samples from the healthy population and patients with CVDs. This leads to the conclusion that high optimism can indirectly reduce the risk of CVDs through healthy behavioral choices [22]. Consistently, a recent longitudinal study [23] reported that higher dispositional optimism at baseline was associated with a greater likelihood of reporting a sustained healthy lifestyle (i.e., physical activity, body mass index, diet, alcohol, and tobacco consumption) over ten years of follow-up in a sample of healthy women. Similarly, another prospective study [24] on a cohort of 1113 adults from the general population examined the independent association between dispositional optimism and pessimism and ideal cardiovascular health metrics, consisting of diet, physical activity, body mass index, smoking status, blood pressure, total cholesterol, and plasma glucose. Findings showed an association between lower pessimism and nonsmoking, healthy diet, and ideal physical activity, while optimism was positively associated with eating a healthy diet. Again, one further study on middle-aged adults found that pessimism was negatively correlated to healthy dietary habits and independently reduced any improvement in dietary patterns [25].

Although extensive literature has shown an association between dispositional optimism, healthy lifestyles, and positive physical health outcomes, some studies have, by contrast, emphasized the detrimental role of excessive optimism. People who are more optimistic perceive that they are at lower risk for a variety of disease-related outcomes [26,27]. For example, Gerend et al. [26] examined a sample of 312 women aged 40–86 and found that more optimistic women perceived that they were at lower risk for breast cancer, heart disease, and osteoporosis. Hamilton and Lobel [27] confirmed this finding in a sample of 452 younger (ages 18–25 years) and 167 middle-aged (ages 40–64 years) women, showing that more optimistic women perceived lower risk of CVDs and breast and lung cancer. Importantly, prior literature has shown that lower cardiovascular risk perception may negatively influence the implementation of preventive actions (including cardiovascular screening, physical activity, diet, and smoking) to reduce CVDs [28].

The sense of coherence (SOC) defines a way of thinking that enables people to identify and use the resources that are available to them: the more an individual can understand (comprehensibility), handle (manageability), and make sense of (meaningfulness) a stressful situation or disease, the greater his/her potential to cope with it successfully [20]. Prior studies have shown that a strong SOC is associated with better-perceived health and lower rates of CVD and mortality [29]. Associations between higher SOC scores and healthy lifestyles have commonly been reported among the healthy population [30,31] and individuals with cardiovascular risk [32]. For example, Binkowska-Bury et al. [30] explored lifestyles in a sample of Polish individuals living in rural areas and showed that people with a higher SOC smoked less. Wainwright et al. [31] performed a cross-sectional study focused on 18,287 adult residents of the United Kingdom and found that individuals with a strong SOC were less likely to be active smokers, more likely to be physically active, and reported healthier nutrition behaviors. An exception was that people with a strong SOC consumed more alcohol. A further prospective population-based cohort study [33] demonstrated that, based on a mean follow-up of 8 years, higher levels of the SOC were associated with a 20% reduced risk of all-cause mortality. Notably, socioeconomic status and lifestyle choices (i.e., cigarette smoking, physical activity, dietary intakes of fruit, vegetables, and fiber) explained this association. Moreover, a recent study [34] on a middle-aged population explored the relationship between multiple health risk behaviors and specific psychosocial factors, including the SOC; the authors showed that a low SOC was associated with insufficient physical activity and risky alcohol intake.

As previously mentioned, behaviors such as physical activity, diet, alcohol, and tobacco consumption tend to cluster in the healthy population [35], leading to a lifestyle that can have a multiplying effect on cardiovascular health compared to individual behaviors [36]. Therefore, studying the interplay among healthy behaviors and identifying different lifestyle profiles are crucial and may play a central role in the primordial prevention of CVDs. Although the previously cited studies demonstrated an association between dispositional optimism, SOC, and different health-related behaviors, it remains unclear whether dispositional optimism and the SOC are associated with the adoption of multiple concurrent health-related behaviors, which synergistically impact cardiovascular health. To date, the literature investigating the clustering of health behaviors in the healthy population is vast, but there are few studies reporting their associations with physical health outcomes [36,37], and there are even fewer that provide insight into the influence of psychological factors such as dispositional optimism and the SOC [38], especially when considering the sub-dimensions of the latter. These sub-dimensions refer to different psychological domains, namely cognitive (comprehensibility), affective (meaningfulness), and behavioral (manageability) [39,40], and could play a different role in health-related behaviors. Interestingly, a study focused on the smoking behaviors of pregnant women found that women who relapsed to smoking showed weaker manageability than women who did not relapse [41].

Starting from all these considerations, we adopted a person-centered approach in the present study [42] to assess how different health-related behaviors can combine to form different lifestyle profiles. The person-centered approach groups individuals based on their similarities in a series of characteristics. It, therefore, allows researchers to explore the functioning of individuals from a more integrated perspective than the more traditional approaches centered on variables, which consider components of the individual taken in isolation [42].

According to this approach, this study aimed to (i) identify different lifestyle profiles, which simultaneously took into account different health-related behaviors, namely diet, alcohol consumption, physical activity, cigarette smoking behavior, and for the first time, the tendency to spontaneously undergo cardiovascular screening; (ii) analyze the association between dispositional optimism, SOC, and health-related lifestyle profiles in a sample of healthy Italian adults. Based on prior research, we hypothesized that (i) higher levels of dispositional optimism and (ii) higher levels of the SOC would be associated with healthier lifestyles. No specific hypotheses were advanced regarding the associations among specific lifestyle profiles and the associated psychological variables due to the paucity of prior literature. Additionally, the main sociodemographic variables (i.e., age, gender, education, and family history for CVDs) were considered in the present work. This choice was driven by prior research suggesting gender, age, and education as relevant indicators shaping people’s lifestyle profiles. In their systematic review, Noble et al. [9] showed that male gender and lower levels of education had the most consistent relationship with riskier clusters. The role of age was not clear, as some studies showed an association between youth and riskier clusters, while others reported the opposite patterns or non-informative results. The systematic review of Meader et al. [8] provided fewer clear results regarding gender than the previous systematic review. However, it confirmed the relationship between lower levels of education and riskier clusters and the lack of clarity of results in the literature regarding age. Regarding the family history of CVDs, as far as we know, no prior research has considered its role in shaping people’s lifestyle profiles.

## 2. Materials and Methods

### 2.1. Participants and Procedure

The data were collected between November 2018 and May 2020 via an anonymous online survey using a snowball sampling method. The volunteers were recruited among the researchers’ pool of acquaintances or through word of mouth and online social networks. The online survey was created, piloted, and then administered using Google Forms. It collected information about sociodemographic indicators, as reported in Table 1, and lifestyles, namely diet, alcohol consumption, physical activity, cigarette smoking behavior, and cardiovascular screening. Lifestyles were assessed following the criteria provided by the guidelines for cardiovascular prevention of Pedretti et al. [43] and Volpe et al. [44]. Two validated questionnaires were included in the online survey to evaluate dispositional optimism and sense of coherence.

A total of 678 participants took part in the online survey. Eligible participants were healthy adults within the 18–60 age range with sufficient Italian language skills. Individuals suffering from a chronic disease or undergoing drug therapy were excluded from participation in the study to evaluate lifestyles with a view to prevention.

Before running further analyses, we screened the data for the presence of multivariate outliers [45] in lifestyle variables. Among participants who completed the questionnaire, two were identified as multivariate outliers and excluded from the analyses.

The sample size adequacy was established by resorting to power analysis [46], using G*Power Version 3.1.9.7 [47]. We calculated the sample size required to perform a two-tail logistic regression with the following parameters: odds ratio = 1.68 (indicates a small effect size according to Chen et al.) [48], α = 0.05, power = 0.95. The sample size calculated was 311 individuals. Based on these considerations, the sample size of the study was sufficient to detect small size effects.

The study received the approval of the Ethics Committee of the authors’ university, and all methods were performed following the relevant guidelines and regulations.

### 2.2. Sociodemographic Indicators

The first part of the online survey collected information about sociodemographic indicators. Besides age, gender, and education, participants were asked to indicate whether they have or have had direct relatives with diseases attributable to cardiovascular disorders (for example, hypertension, acute coronary event, or stroke). This last question was used to indicate family history of CVDs, and it was classified as present vs. not present.

### 2.3. Lifestyle Measures

#### 2.3.1. Diet

To evaluate dietary routines, participants were asked to report the frequency of consumption of three different types of dietary fats (extra virgin olive oil, butter, other fats) through a 6-point Likert scale, where 1 meant “Never” and 6 meant “Every day.” In addition, the participants were asked if they paid attention to their salt consumption through a 3-point Likert scale, where 1 meant “I have never paid attention” and 3 meant “I have always paid attention.” The choice to focus on dietary fats and salt consumption was guided by the evidence that these are two food groups for which a good percentage of the Italian population shows inappropriate behavior [49]. Therefore, they were capable of discriminating between adherent vs. non-adherent individuals.

In order to obtain a concise measure of adherence to international guidelines (https://www.who.int/news-room/fact-sheets/detail/healthy-diet, accessed on 24 October 2021) for use in subsequent analyses, the fat consumption scores were recoded so that a higher score reflected high adherence. Scores for fat and salt consumption were then standardized and averaged. The participants who reported consuming extra virgin olive oil daily and other dietary fat (i.e., butter, margarine) less than once a week and who declared that they had decreased or had always been attentive to their salt consumption were considered adhering to a healthy diet, according to the national guidelines (https://www.salute.gov.it/portale/documentazione/p6_2_2_1.jsp?lingua=italiano&id=2915, accessed on 24 October 2021).

#### 2.3.2. Alcohol Consumption

To evaluate total alcohol intake, participants were asked to report how often they consumed beer, wine, and spirits through a 3-point Likert scale, where 1 indicated “A teetotaler (never drink alcohol—wine, beer or spirits),” 2 indicated “An occasional drinker (drink on occasion but not every day),” and 3 indicated “A regular drinker (drink alcohol daily).” This classification was based on previous studies [50].

In order to obtain a concise measure of adherence to guidelines for a healthy lifestyle for use in subsequent analyses, the alcohol consumption scores were recoded so that a higher score reflected high adherence. The participants who abstained or reported drinking occasionally were considered to adhere to a healthy lifestyle.

#### 2.3.3. Physical Activity

Physical activity was measured using the Rapid Assessment of Physical Activity Questionnaire-1 (RAPA-1) [51]. It is a seven-item questionnaire that assesses the type and amount of physical activity reported by the participant through dichotomous queries and assigns a final score from 1 (i.e., absence of physical activity) to 7 (i.e., vigorous physical activity).

A higher score reflected higher adherence to guidelines for a healthy lifestyle. A final score of 6 or 7 was considered adequate.

#### 2.3.4. Cigarette Smoking Behavior

According to previous research [52], one item was used to assess participants’ smoking behavior: “Do you currently smoke?”. Answers were on a 5-point Likert scale where 1 meant “Yes, I currently smoke” and 5 meant “No, I have never smoked.”

A higher score reflected higher adherence to guidelines for a healthy lifestyle. The participants who have never smoked or declared that they had quit at least a year before were classified as adherents to a healthy lifestyle.

#### 2.3.5. Cardiovascular Screening

Participants had to report whether they had undergone cardiovascular check-ups and give the reason why or why not. Due to the paucity of prior studies on this aspect, an ad-hoc question was created (i.e., “Have you ever had cardiovascular screening tests to check your health? For what reason did you have these checks?”). Responses were rated on a 5-point Likert scale, where 0 meant “I have never had a cardiovascular screening,” 1 meant “Obligation (i.e., medical certificates for physical activity, exams required in the workplace),” 2 meant “Both by obligation and by personal will,” 3 meant “Will/personal interest.”

A higher score reflected higher adherence to guidelines for a healthy lifestyle. The participants who declared that they had undergone cardiovascular screening out of will and not only out of obligation were classified as adherents to a healthy lifestyle.

### 2.4. Psychological Measures

#### 2.4.1. Dispositional Optimism

This variable was measured using the brief Italian version of the Life Orientation Test (LOT-R) [53,54]. This questionnaire is composed of six items that assess dispositional optimism. Items are scored on a 5-point Likert scale (from 1 = strongly disagree to 5 = strongly agree), with higher total scores indicating greater dispositional optimism. An example item is “I am always optimistic about my future.” The internal consistency of the scale in the present sample was adequate (Cronbach’s alpha = 0.81).

#### 2.4.2. Sense of Coherence

The Sense of Coherence Scale (SOC) [39,55] is a 13-item self-report measure of how people manage stressful situations and stay well (i.e., the sense of coherence). The SOC scale is composed of three subscales, namely comprehensibility (five items, for example: “Do you have the feeling that you are in an unfamiliar situation and do not know what to do?”), manageability (four items, for example: “How often do you have feelings that you are not sure you can keep under control?”), and meaningfulness (four items, for example: “How often do you have the feeling that there is little meaning in the things you do in your daily life?”), which can be added to a total score. Higher scores indicate higher levels of comprehensibility, manageability, and meaningfulness. All the answers were given on a 7-point Likert scale, on which the alternatives were semantically different and ranged from 1 “very seldom or never” to 7 “very often.” The scale showed a discrete internal consistency in the present sample (Cronbach’s alpha ranged from 0.66 for the meaningfulness subscale to 0.84 for the total score).

### 2.5. Data Analysis

Data analyses were performed with SPSS, version 26, Jamovi, version 1.6, and specific modules of Sleipner [56], a statistical package for typological analyses.

Cluster analyses were performed on the continuous scores of the five lifestyle variables. We followed the suggestion of Bergman [57]. Firstly, all five lifestyle variables were z-standardized. Moreover, according to standard options [58], a residue analysis was performed (i.e., average squared Euclidean distance—ASED—less than 0.5). As a result, two multivariate outliers were identified and excluded from the subsequent analyses. Then, a two-step clustering procedure was applied to typify participants depending on their behaviors. First, results from Ward’s hierarchical method, followed by the non-hierarchical k-means method, were combined. Various solutions were chosen based on the size of the change in the error sum of squares (ESS) value between adjacent cluster solutions in the hierarchical method. Therefore, each solution was used as the initial cluster center for a non-hierarchical k-means clustering procedure. After this non-hierarchical clustering method, five indices were used to evaluate the optimal number of clusters to extract: C-index, G (+) index, W/B index, gamma index, and point-biserial correlation. The minimum value of the former three indices and the maximum of the latter two suggested the optimal number of clusters to retain, hence the best cluster solution. Another criterion for cluster solution retention was a reasonable cluster size (i.e., every cluster contained at least 5% of all the cases) [59].

A multinomial logistic regression analysis was performed, with the variable “lifestyle profile” as the dependent variable (5 levels, one for each cluster identified by the typological analysis) and the psychological variables (dispositional optimism and the three dimensions of SOC scale) as covariate predictors. In addition, demographic variables, namely gender, age, educational level (less than high school vs. high school or higher), and family history of cardiovascular disease (yes vs. no), were included as categorical predictors (gender, educational level, family history of cardiovascular disease) or covariate (age) in the regression analysis to assess their potential role in determining the probability of belonging to a specific profile.

## 3. Results

### 3.1. Study Sample

The final sample consisted of 676 participants, 313 males (46.3%), with a mean age of 35 (SD = 11.6, range 19–57). A detailed description of the sociodemographic characteristics of the sample is reported in Table 1.

### 3.2. Identification of Lifestyle Profiles

**Figure 1:**
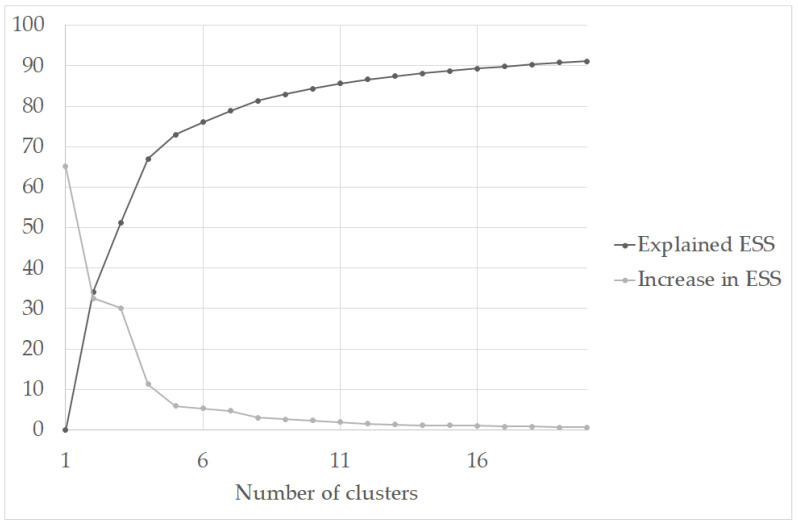
Error sum of squares plot for the activity cluster solution. ESS = error sum of squares.

After evaluating the scree-type plot (Figure 1) showing the change in the ESS by cluster solutions and based on the size of the change in the ESS values, the solutions from the three to five clusters were retained for further analysis. Table 2 presents the fit indices of the retained cluster solutions. Even though the point-biserial correlation and gamma index are higher, and the G+ index is lower in the three-cluster solution, the other indices are more appropriate in the five-cluster solution. Moreover, the five-cluster solution showed a much higher explained ESS than the three-cluster solution and more reasonable cluster sizes. Collectively, these considerations identified the five-cluster solution as the optimal one.

**Figure 2:**
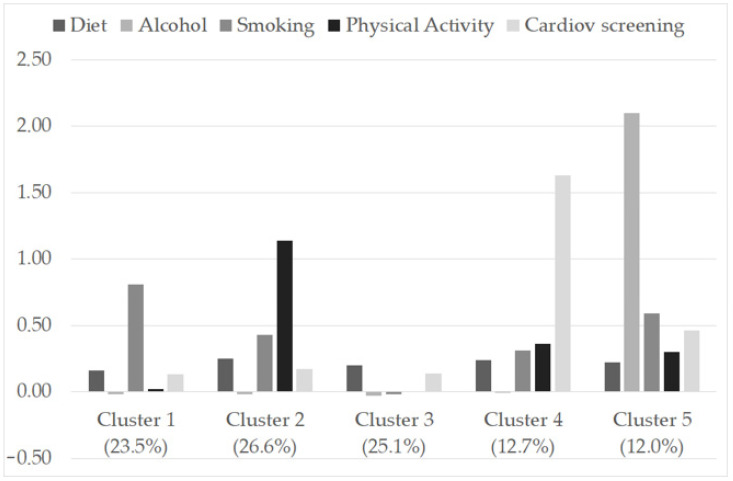
Z-scores for diet, alcohol consumption, cigarette smoking, physical activity, and cardiovascular screening for the final five-cluster solution.

Figure 2 presents the final cluster solution. The *Y*-axis represents z-scores. Because the clusters were defined using z-scores for the total sample, each cluster’s mean z-scores indicate the distance between the cluster means and the total sample’s standardized mean. In other words, Z-scores between −0.5 and +0.5 denote an average value (i.e., the “average participant” lifestyle). A Z-score over +0.5 denotes values above the sample mean (i.e., healthier than the average lifestyle) and thus higher adherence to a healthy diet, lower alcohol consumption, less smoking, higher physical activity, and higher cardiovascular prevention than the “average participant.” Table 3 reports a sociodemographic description and adequateness of lifestyle for the clusters identified.

Cluster 1 was made up entirely of individuals who were adherent to international guidelines regarding smoking; in other words, 100% of the individuals belonging to this cluster had never smoked or had quit at least one year before. This cluster also adhered highly to international guidelines regarding alcohol consumption, as 90% of the individuals belonging to this cluster did not drink or reported drinking occasionally. Regarding the remaining behaviors, this cluster had the lowest adherence to international guidelines regarding diet and physical activity (see Table 3). Notably, none of the individuals belonging to this cluster performed an adequate amount of physical activity, and only 23% adhered to international guidelines by paying attention to how much dietary fats and salt they consumed.

Cluster 2 was markedly distinguished from the other clusters due to their adherence to international guidelines regarding physical activity. One hundred percent of the individuals belonging to this cluster performed adequate physical activity, compared to very low percentages in the other clusters.

Respondents fitting cluster 3 exhibited “average behavior” across all five lifestyles (see Figure 2). None of the individuals belonging to this cluster performed an adequate amount of physical activity. This cluster was also the lowest to adhere to international guidelines regarding alcohol (which in any case was high, around 86%) and tobacco consumption (only 29% of individuals had never smoked or had quit more than a year before).

Cluster 4 included individuals who were sensitive to cardiovascular prevention, considering that 100% of them underwent cardiovascular screening by will and not only for reasons dictated by the circumstances.

Finally, cluster 5 was entirely made up of individuals who were adherent to the international guidelines regarding alcohol consumption. This cluster also included the highest percentage of individuals who watched their consumption of dietary fats and salt, thus adhering to international guidelines, although this percentage was low (38%) and similar to that observed in clusters 2 (37%) and 4 (37%).

### 3.3. Associations between Lifestyle Profile, Sociodemographic, and Psychological Variables

The multinomial logistic regression results indicate that the whole model containing all predictors was statistically significant (χ^2^(32, N = 676) = 88.68, *p* < 0.001), indicating that the model could distinguish among respondents from the different lifestyle profiles. The model explained between 12.3% (Cox and Snell’s R^2^) and 13% (Nagelkerke’s R^2^) of the variance from belonging to one of the lifestyle profiles identified. As shown in Table 4, five independent variables made a unique, statistically significant contribution to the model (age, gender, SOC Comprehensibility, SOC Manageability, and optimism). Cluster 3 was chosen as the reference level for comparisons between clusters, considering that respondents who fit this profile exhibited “average behavior” across all five lifestyles (see Figure 2).

The most robust predictor of lifestyle profile was gender. The statistically significant contrast between clusters 2 and 3 (*p* < 0.001) and the corresponding odds ratio (2.40) indicated that men were two times more likely than women to belong to the lifestyle profile that engaged in more physical activity while controlling for all other factors in the model. Similarly, the statistically significant contrast between clusters 4 and 3 (*p* = 0.010) and the corresponding odds ratio (2.09) indicated that men were two times more likely than women to belong to the lifestyle profile characterized by greater attention to cardiovascular screening.

Results show that older participants were more likely than younger participants to belong to the lifestyle profile that paid greater attention to cardiovascular screening (*p* < 0.001) and the one with lower alcohol consumption (*p* < 0.001). For every unit increase in age, participants were more likely to belong to the lifestyle profile characterized by greater attention to cardiovascular screening and lower alcohol consumption. The odds ratios were close to 1 (both 1.05), suggesting a very small effect size of this variable.

Neither education nor family history of CVDs significantly determined the likelihood of belonging to the different lifestyle profiles.

The role of dispositional optimism emerged in the contrast between clusters 4 and 3 (*p* = 0.043). The odds ratio for dispositional optimism was less than 1 (0.67), indicating that, for every unit increase in optimism, participants were 0.67 less likely to belong to the lifestyle profile characterized by greater attention to cardiovascular screening.

Finally, the role of the SOC emerged in the contrast between clusters 1 and 3 (*p* = 0.027 for the comprehensibility dimension and *p* = 0.020 for the manageability dimension). The odds ratio for comprehensibility was less than 1 (0.67), indicating that, for every unit increase in comprehensibility, participants were 0.67 less likely to belong to the lifestyle profile characterized by unhealthy diet, sedentary behavior, and nonsmoking. Conversely, the odds ratio for manageability showed that participants were 1.43 times more likely to belong to this lifestyle profile for every unit increase in the manageability score.

## 4. Discussion

This study explored the associations between sociodemographic indicators, dispositional optimism, SOC, healthy lifestyle, and CVD preventing behaviors in a sample of healthy Italian adults, adopting a person-centered approach.

Five clusters were identified, and each was characterized by its unique and specific profile of lifestyle combinations. Overall, the configuration of the clusters that emerged confirmed the grouping of two types of behaviors suggested by the prior literature [8,9,60]. The first grouping concerns smoking and alcohol behaviors, which can be conceived as addictive behaviors requiring restraint or abstinence [9,60]. In the present study sample, these behaviors tend to cluster both in terms of high adherence to international guidelines (clusters 1 and 5) and in terms of low adherence (cluster 3). The second grouping concerns physical activity and dietary fat and salt consumption, which can be conceived as behaviors that require an active commitment to promoting one’s health [9,59]. These behaviors tend to group, albeit weakly, in terms of great adherence to international guidelines (clusters 2, 4, and 5) and low adherence (clusters 1 and 3).

Regarding the sociodemographic variables associated with the lifestyle profiles identified, a very small effect emerged for the association between older age and the lifestyle profiles characterized by great attention to cardiovascular screening and low alcohol consumption. More interestingly, informative associations with gender were found. Men were much more likely than women to belong to the lifestyle profile with the highest physical activity (cluster 2) and the greatest attention to cardiovascular screening (cluster 4). The first result is coherent with prior literature highlighting gender disparity in interest and participation in physical activity. For example, Riera-Sampol et al. [32] found that a large sample of Spanish men with cardiovascular risk factors reported more physical activity than women with the same clinical characteristics. A recent review [61] showed that men tend to be more physically active than women throughout the life cycle and that these differences may be related to different motivations for individuals to exercise. For men, the motivations tend to be more intrinsic, such as improving health, preventing non-communicable diseases, improving body shape, and being competitive. For women, the motivations are intrinsic and extrinsic and include social aspects, a sense of well-being, and a positive body image. Sciomer et al. [62] also underlined that women living in so-called high-income countries have multiple roles—working and managing their families—which take time and energy and leave little room for leisure activities.

The second finding is novel and particularly interesting. Men’s greater tendency to undergo cardiovascular screening suggests that men may be more aware of the cardiovascular risks they run. One possible explanation is the general idea that CVDs are more common in men than in women. Although this idea has a scientific basis [63], it has been demonstrated that women have a higher rate of mortality and poorer prognosis following an acute cardiovascular event [64]. CVD risk in women is often underestimated due to the misperception that women are more “protected” than men against CVDs [6,65]. However, it is essential to consider cardiovascular risk factors particular to women, which manifest at a young age and have long-term effects. Among them are the physiological reduction of estrogen with age, which evolves slowly and many years before the codified time of menopause, hypertensive disorders of pregnancy, and gestational diabetes, the effects of which are long-lasting [44,66]. Healthy lifestyle approaches are fundamental in countering these long-term risk factors [62]. Overall, our results suggest the need for raising awareness among the healthy female population about cardiovascular risk and the usefulness of engaging in regular physical activity in their leisure time.

There was no association between education and lifestyle profile in this study. This may be a result of the high level of education of the sample (54% of the respondents had at least a bachelor’s degree) compared to previous studies [8,9]. The decision to select a sample of healthy adults, therefore relatively young (in the range of 18–60 years), could have produced this result. Our result allows us to speculate about the future. Considering that new generations are on average more educated than past generations, the role of education on lifestyles may fade over time.

Similarly, our results show no association between family history of CVDs and lifestyle profile. This may be a result of the high percentage of respondents (73%) who claimed to have a direct relative with diseases attributable to cardiovascular origins and therefore on the low discriminating capacity of this variable. It is helpful to underline how our sample’s high familiarity with CVDs also confirms the high incidence of CVDs in the Italian population [2].

Regarding the association between positive psychological resources and lifestyle profiles, the results show that the participants with lower dispositional optimism scores were more likely to belong to the lifestyle profile paying the greatest attention to cardiovascular screening (cluster 4). At first glance, this result seems to contrast with the previously cited studies suggesting that higher optimism levels are related to a greater likelihood of engaging in healthy behaviors [22,23], and it refutes our original hypothesis. However, this result did not emerge in the clusters characterized by the healthy behaviors typically investigated in the previous studies. It emerged in the cluster with the highest adherence to the guidelines concerning cardiovascular screening or, in other words, in the cluster in which all individuals declared that they undergo cardiovascular screening not only out of obligation but out of will (see Table 3). Therefore, the findings suggest that people with lower optimism scores voluntarily undergo cardiovascular screening more frequently than those with higher optimism scores. This highlights a downside to optimism and suggests that individuals with high dispositional optimism may underestimate cardiovascular risk. Coherently, prior works have shown that more optimistic people perceive that they are at lower risk for various disease-related outcomes [26,27], thus underestimating any adoption of unhealthy behaviors [28].

The results show an association between the likelihood of belonging to cluster 1 and two dimensions of the SOC, namely manageability and comprehensibility. The participants characterized by higher scores in the manageability subscale were more likely to belong to this lifestyle profile. This result goes in the same direction as prior findings indicating that a strong SOC is associated with lower percentages of cigarette smoking [33,67]. Let us recall that this cluster had the highest adherence to healthy behavior regarding nonsmoking and a high adherence regarding low/no alcohol consumption and that these two behaviors can be conceived as addictive, thus requiring restraint or abstinence [9,60]. Thus, our result suggests that individuals with strong manageability are less likely to develop addictions to alcohol and smoking. Indeed, manageability refers to the instrumental or behavioral dimension of the SOC and denotes the feeling of exercising specific control over events or stressful situations. It represents the extent to which an individual perceives her/his internal and external resources as available and adequate to handle the demands posed by stimuli from one’s internal and external environment [39]. Interestingly, a study focused on the smoking behaviors of pregnant women found that women who relapsed to smoking showed a weaker SOC than women who did not relapse, particularly in the manageability component [41].

The results also show that the participants with higher scores in the comprehensibility SOC subscale were less likely to belong to cluster 1. Reasons for this finding could be sought in the fact that this cluster was characterized not only by nonsmoking and low alcohol consumption but also by the lowest physical activity, the unhealthiest diet, and the lowest attention to cardiovascular screening. As already mentioned, these are behaviors that tend to cluster and require an active commitment to promoting one’s health [9,60]. Comprehensibility refers to understanding what happens in one’s surroundings and classifying events in a specific family and social framework [40]. It refers to the cognitive dimension of the SOC, and it expresses the extent to which an individual perceives internal and external stimuli as understandable, rational, coherent, and structured. This attribute helps individuals better understand the context in which they live and more easily delineates their role in it. Lower comprehensibility could therefore lead to a poor understanding of the importance of actively promoting one’s health, resulting in the adoption of inappropriate health-related behaviors.

Overall, the results regarding the SOC show that its sub-components may play different roles in profiling healthy lifestyles and therefore suggest that they should be considered separately in future studies on this research area.

This study presents some limitations that should be acknowledged. First, the observational and cross-sectional nature of the study does not allow for the establishment of a causal association between dependent and independent variables. Second, all information on lifestyles and psychological variables was self-reported and thus prone to information bias such as recall bias or social desirability bias. Indeed, the brief self-report questionnaires used to evaluate psychological variables could complicate assessing these complex psychological constructs. The same limit could be mentioned regarding the self-report questionnaires used to assess lifestyles. This approach may restrict the results’ reliability because participants may have over- or underestimated their real lifestyle. More ecologically valid methods might be helpful to evaluate the truthfulness of individuals’ reported information. Regarding this last impediment, although methodological and inferential limitations constrain self-reported measures, they are suitable to provide essential steps in understanding a phenomenon [68], and they have substantial advantages, such as ease of use and excellent cost–benefit ratio. Moreover, it must be noted that the diet assessment, which is based on the national guidelines, was adapted to the Italian cultural background and this may have reduced the generalizability of the findings to other countries. Despite this, it is noteworthy that the Mediterranean diet is likewise recognized internationally, thus allowing for the possible replicability of results in a different context. Finally, we used an online survey and enrolled participants through a snowball sampling method. Therefore, the present convenience sample may be unrepresentative of the target population of healthy Italian adults.

Despite limitations, this study reported original findings that suggest important implications for further research and practice. It identified distinct profiles of health-related behaviors within a large sample. The exploration of multiple co-occurrent health-related behaviors and the person-centered analytical approach allowed us to investigate the functioning of individuals from a more integrated perspective than the more traditional approaches centered on variables and represents the main strength of this study. Information about whether and which health-related lifestyles cluster together can facilitate identifying vulnerable population groups for targeting health promotion strategies and contribute to developing effective and holistic preventive health interventions [9]. Because of the possible synergistic effects of multiple health-related behaviors [36], multiple behavior change interventions have a potentially more significant impact on public health than interventions aimed at single risk factors.

## 5. Conclusions

Overall, the results of the study suggest that women need to be more motivated to engage in regular physical activity and undergo cardiovascular screening to promote cardiovascular health. More efforts need to be made to raise women’s awareness of cardiovascular risk. Furthermore, the results of the study suggest, contrary to what might be hypothesized, that individuals with higher dispositional optimism require attention because they may underestimate cardiovascular risk. Incorrect optimism could be managed by supporting individuals to raise their awareness about the actual cardiovascular risk they run. Finally, the results suggest the importance of the SOC in sustaining a healthy lifestyle. Strong manageability could play a protective role in behaviors requiring restraint or abstinence, such as smoking and alcohol consumption. Weak comprehensibility could negatively affect behaviors that require an active commitment to promoting one’s health, such as physical activity, a healthy diet, and cardiovascular screening. Manageability might be influenced by helping people find resources to manage stressful events without using smoking or alcohol consumption as coping strategies. Comprehensibility could be increased by supporting individuals to raise their awareness about the importance of active commitment in health-promoting behaviors.

In a primordial prevention framework, the promotion of healthy behaviors in the adult population should consider individuals’ psychological characteristics, including dispositional optimism and SOC. Targeting such factors along with correct lifestyles through the adoption of a clustering approach should be supported and may represent a key strategy to counteract the risk factors and pre-empt the premature onset of diseases. Following this line, future steps may, for example, consist of the implementation of innovative technological tools (e.g., smartphone apps) making it possible to profile individuals and the consequent tailored communication of health claims. As suggested by prior works, mobile-app-based health promotion strategies have already shown their usefulness [69].

## Figures and Tables

**Table 1:** Sociodemographic characteristics of the sample (*n* = 676).

Sociodemographic Variables	
Age, mean (SD)	34.7 (11.6)
Gender, *n* (%)	
Female	363 (53.7%)
Male	313 (46.3%)
Educational level, *n* (%)	
high school or less	310 (45.9%)
higher than high school	366 (54.1%)
Family history of cardiovascular disease	
yes	492 (72.8%)
no	184 (27.2%)

**Table 2:** Fit indices of the three- and five-cluster solution identified through K-means cluster analysis.

Goodness-of-Fit Indices	3-Cluster	5-Cluster
Explained ESS	51.2095	72.9547
Point-biserial correlation	0.7850	0.5007
C-index	0.2028	0.1007
Gamma index	0.9272	0.8658
W/B index	0.2718	0.1968
G+ index	0.0175	0.0229

**Table 3:** Sociodemographic description and adequateness of lifestyle for the clusters identified.

Cluster	N (%)	Mean Age (SD)	% Male	% Healthy Lifestyles				
				Diet	Alcohol Consumption	Physical Activity	Cigarette Smoking	Cardiovascular Screening
1	159 (23.5%)	33.6 (11.8)	43.4%	23.3%	89.9%	0.0%	100.0%	50.9%
2	180 (26.6%)	33.0 (10.7)	57.8%	36.7%	90.0%	100.0%	68.9%	58.9%
3	170 (25.1%)	33.1 (11.0)	38.8%	31.8%	86.5%	0.0%	28.8%	52.4%
4	86 (12.7%)	38.9 (12.1)	55.8%	37.2%	94.2%	32.6%	53.5%	100.0%
5	81 (12.0%)	39.0 (11.9)	32.1%	38.3%	100.0%	29.6%	82.7%	70.4%

Note. In this table, we dichotomized each behavior according to whether participants met recommended guidelines. Percentages of adherent participants are reported. Regarding the diet, participants who stated that they consumed extra virgin olive oil daily and other dietary fat (i.e., butter, margarine) less than once a week and who declared that they had decreased or had always been attentive to their salt consumption were considered as adhering to a healthy diet, according to the national guidelines. Regarding alcohol consumption, participants who abstained or reported drinking occasionally were considered to adhere to a healthy lifestyle. Regarding physical activity, participants who obtained a score of 6 or 7 on RAPA-1 were classified as adherents. As regards smoking, participants who have never smoked or declared that they had quit at least a year before were classified as adherents. Finally, concerning cardiovascular prevention, participants who declared that they had undergone cardiovascular screening out of will and not only out of obligation were classified as adherents.

**Table 4:** Multinomial logistic regression analyzing influences of the demographic and psychological variables on lifestyle profile.

							95% CI	
Cluster	Predictor	Estimate	SE	Z	*p*	OR	Lower	Upper
1–3	Intercept	−0.69	0.68	−1.02	0.306	0.50	0.13	1.88
	Age	0.01	0.01	0.57	0.570	1.01	0.99	1.03
	Gender	0.24	0.24	1.02	0.308	1.27	0.80	2.03
	Education	0.01	0.24	0.04	0.971	1.01	0.64	1.60
	Family history of CVD	−0.13	0.26	−0.50	0.615	0.88	0.53	1.46
	Optimism	−0.15	0.16	−0.92	0.360	0.86	0.63	1.18
	SOC: Comprehensibility	−0.40	0.18	−2.21	0.027	0.67	0.47	0.96
	SOC: Manageability	0.36	0.15	2.33	0.020	1.43	1.06	1.93
	SOC: Meaningfulness	0.22	0.13	1.70	0.090	1.25	0.97	1.61
2–3	Intercept	−1.52	0.68	−2.23	0.026	0.22	0.06	0.83
	Age	−0.00	0.01	−0.36	0.722	1.00	0.98	1.02
	Gender	0.87	0.23	3.77	<0.001	2.40	1.52	3.78
	Education	0.07	0.23	0.28	0.777	1.07	0.68	1.68
	Family history of CVD	0.10	0.24	0.43	0.667	1.11	0.69	1.79
	Optimism	0.26	0.16	1.66	0.096	1.30	0.95	1.78
	SOC: Comprehensibility	−0.30	0.18	−1.72	0.086	0.74	0.52	1.04
	SOC: Manageability	0.12	0.15	0.83	0.407	1.13	0.85	1.51
	SOC: Meaningfulness	0.22	0.13	1.72	0.085	1.25	0.97	1.60
4–3	Intercept	−2.24	0.80	−2.80	0.005	0.11	0.02	0.51
	Age	0.05	0.01	3.93	<0.001	1.05	1.02	1.08
	Gender	0.74	0.28	2.59	0.010	2.09	1.20	3.65
	Education	0.17	0.29	0.59	0.552	1.18	0.68	2.07
	Family history of CVD	0.10	0.31	0.34	0.732	1.11	0.61	2.03
	Optimism	−0.40	0.20	−2.02	0.043	0.67	0.46	0.99
	SOC: Comprehensibility	−0.29	0.22	−1.33	0.182	0.75	0.49	1.15
	SOC: Manageability	0.21	0.18	1.13	0.257	1.23	0.86	1.77
	SOC: Meaningfulness	0.21	0.15	1.36	0.175	1.23	0.91	1.67
5–3	Intercept	−1.24	0.81	−1.54	0.124	0.29	0.06	1.40
	Age	0.05	0.01	3.78	<0.001	1.05	1.02	1.07
	Gender	−0.34	0.30	−1.13	0.257	0.71	0.40	1.28
	Education	−0.07	0.29	−0.23	0.815	0.93	0.53	1.65
	Family history of CVD	0.02	0.32	0.07	0.943	1.02	0.55	1.91
	Optimism	8.68 × 10^−4^	0.20	0.00	0.997	1.00	0.68	1.48
	SOC: Comprehensibility	−0.40	0.22	−1.83	0.068	0.67	0.44	1.03
	SOC: Manageability	0.23	0.19	1.21	0.227	1.25	0.87	1.81
	SOC: Meaningfulness	−0.05	0.15	−0.30	0.762	0.95	0.71	1.29

## Data Availability

The data presented in this study are available on request from the corresponding author. The data are not publicly available due to privacy and ethical restrictions.
